# Geographic source estimation using airborne plant environmental DNA in dust

**DOI:** 10.1038/s41598-021-95702-3

**Published:** 2021-08-10

**Authors:** Chelsea Lennartz, Joel Kurucar, Stephen Coppola, Janice Crager, Johanna Bobrow, Laura Bortolin, James Comolli

**Affiliations:** 1 MIT Lincoln Laboratory, 244 Wood Street, Lexington, MA 02421 USA; 2 Present Address: Mercy Bioanalytics, 700 Main Street, Cambridge, MA 02139 USA

**Keywords:** Molecular biology, Plant sciences, Environmental sciences

## Abstract

Information obtained from the analysis of dust, particularly biological particles such as pollen, plant parts, and fungal spores, has great utility in forensic geolocation. As an alternative to manual microscopic analysis of dust components, we developed a pipeline that utilizes the airborne plant environmental DNA (eDNA) in settled dust to estimate geographic origin. Metabarcoding of settled airborne eDNA was used to identify plant species whose geographic distributions were then derived from occurrence records in the USGS Biodiversity in Service of Our Nation (BISON) database. The distributions for all plant species identified in a sample were used to generate a probabilistic estimate of the sample source. With settled dust collected at four U.S. sites over a 15-month period, we demonstrated positive regional geolocation (within 600 km^2^ of the collection point) with 47.6% (20 of 42) of the samples analyzed. Attribution accuracy and resolution was dependent on the number of plant species identified in a dust sample, which was greatly affected by the season of collection. In dust samples that yielded a minimum of 20 identified plant species, positive regional attribution was achieved with 66.7% (16 of 24 samples). For broader demonstration, citizen-collected dust samples collected from 31 diverse U.S. sites were analyzed, and trace plant eDNA provided relevant regional attribution information on provenance in 32.2% of samples. This showed that analysis of airborne plant eDNA in settled dust can provide an accurate estimate regional provenance within the U.S., and relevant forensic information, for a substantial fraction of samples analyzed.

## Introduction

The ability to match a person or object with a particular location, known as geographic attribution, can be a crucial part of a forensic investigation. Geographic attribution often relies on the analysis of dust because of its ubiquity, abundance, adherence to most surfaces, and wide variety of component particles that includes bacterial and fungal cells, spores, minerals, soil, plant and animal components, and products of combustion and other human activities^1^. Each of these particle types can be indicative of the local exposure history of the sampled object or person, and their analysis and interpretation has been used for almost a century as a tool in forensic and investigative geographic attribution^2–4^.

Biological components of dust have proven to be particularly valuable as an investigative tool. Pollen has traditionally been the primary information source since it is widespread, stable, and taxonomically diverse in structure so that geographic discrimination based on plant biogeography is possible^5–7^. Since its release varies seasonally, pollen can also provide information on the timing of dust accumulation^8^. Historically, pollen characterization has relied heavily on microscopic evaluation of exine structure^9^, which requires time-consuming pollen preparation and manual analysis. This greatly limits sample testing capacity^10^. In addition, accurate taxonomic identification and geographic inference from pollen images requires substantial technical expertise and, even with that, is difficult for many plant taxa. This has further limited widespread application of the technique for forensic investigation and other applications^11^. A number of approaches have been taken in an attempt to address this shortcoming, including the Pollen Identification and Geolocation Technology (PIGLT) system, a standardized digital database of pollen images for automated characterization^12,13^.

More recently DNA barcoding, a technique developed for taxonomic identification in environmental tracking, biodiversity studies, and product authentication^14–16^, has been applied to pollen classification^17^. Barcoding targets a genomic DNA region that is common among taxa so it can be readily amplified, but whose sequence differs sufficiently to enable discrimination. Taxonomic identification is typically performed by matching a generated barcode sequence to a database of barcode sequences from characterized taxa. Barcodes can be specific to a particular family or genus, but “universal” DNA barcodes have been implemented to help identify taxa in broad phylogenetic categories such as bacteria/archaea, fungi, and animals. Universal barcodes can also be used to analyze mixed samples, termed DNA metabarcoding. Plant DNA metabarcoding is challenged by the lack of truly universal barcode that can effectively discriminate most plant species^18–21^, though several are employed. This includes the chloroplast gene encoding the large subunit of ribulose 1,5-bisphosphate carboxylase gene (*rbcL*), the maturase K gene (*matK*), or the intron region of the chloroplast tRNA gene (*trnL*), as well as the nuclear genome encoded internally transcribed spacer region (ITS2) and others^20^. Using these barcodes, DNA barcoding and metabarcoding have been applied toward analysis of isolated pollens and pollen mixtures for the purposes of allergen identification^22–24^, honey characterization^25–28^, forensic geolocation^17^, pollen quantitation^29^, and other applications^30^.

Metabarcoding of environmental DNA (eDNA), the genetic material found in environmental samples such as soil, water, or air, was developed to assess biodiversity without the need for collection of organismal samples^31^. Recently, metabarcoding of fungal eDNA has been demonstrated to discriminate geographically distinct soil samples and to provide information for unguided geographic attribution^32,33^. Using this technique, fungal species from over 900 citizen-scientist collected dust samples from different U.S. locations were characterized^34^ and used to generate a dataset that enabled the estimation of geographic provenance with a median prediction margin of 230 km^35^. Fungal eDNA metabarcoding, in combination with specialized bioinformatic and deep neural network pipelines, has also been used to estimate the worldwide country of origin from dust samples^36,37^. While this approach has the potential to greatly advance geographic attribution capability, it requires the generation of a geographic fungal eDNA reference dataset. This is also true of application of bacterial 16S barcodes for forensic attribution, which have been used to link soil or other samples to a source location^38,39^. Use of environmental bacterial signatures for geographic attribution is also challenged by the tremendous microbiome diversity at local and wider geographic scales^40,41^.

In this study, we explored whether metabarcoding of plant airborne eDNA in dust samples can be used for rapid, high throughput estimation of sample origin without the need for establishing a specialized geographic reference dataset. Two publicly-available databases, the National Center for Biotechnology Information (NCBI) Genbank and the Biodiversity in Service of Our Nation (BISON) data repository, were used for species identification of plant eDNA barcodes and for estimation of the geographic plant species distribution. Our pipeline is intended to provide a less resource intensive alternative to pollen isolation and characterization to gather geographic attribution information from trace samples. Also, since airborne plant eDNA has been shown to capture both anemophilous (wind-pollinated) and non-anemophilous plant species^42^, this approach has the potential to gather source location data from pollen as well as other plant sources such as flower and leaf fragments, spores, and free-floating DNA.

## Results

### Characterization of plant DNA from dust

**Figure 1:**
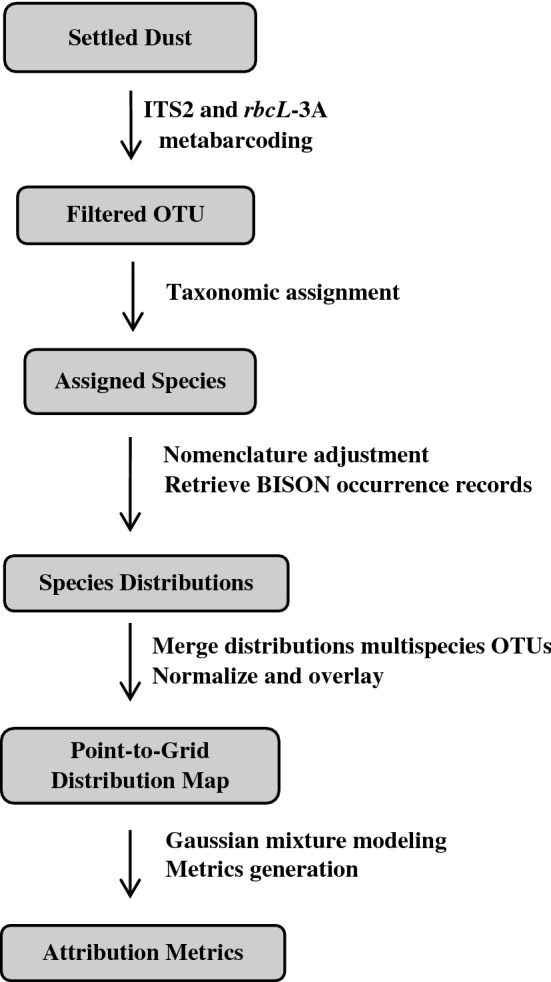
Diagram summarizing the plant eDNA geographic attribution pipeline used in this study. Starting from settled dust, DNA is extracted then subjected to metabarcoding with ITS2 and *rbcL*-3A, sequencing, and data processing to obtain an estimate of the site of origin.

We focused on plants because of the extensive, available barcode sequence and species distribution data. Though a greater diversity of bacterial and fungal DNA barcodes could be generated with most dust samples, the lack of matching sequence and species distribution data limited their utility using our methodology. Our pipeline for estimating geographic origin (summarized in Fig. 1) was initially tested using total airborne plant eDNA isolated from settled environmental dust collected from four distinct U.S. locations.

**Figure 2:**
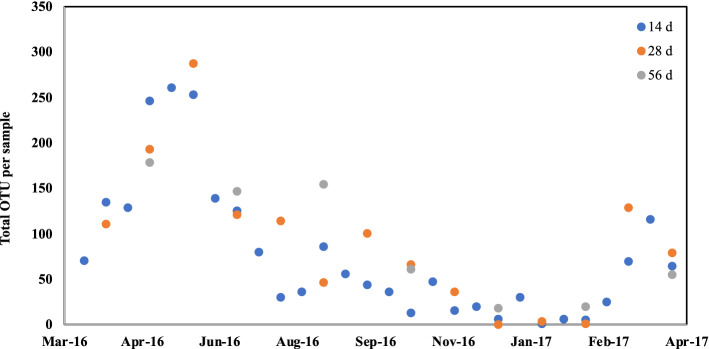
Number of total OTU per sample generated with ITS2 and *rbcL*-3A minibarcodes from dust samples collected in Lexington, MA. Samples were collected on the date indicated after 14 (blue), 28 (orange), or 56 (gray) days of environmental exposure prior to analysis.

**Figure 3:**
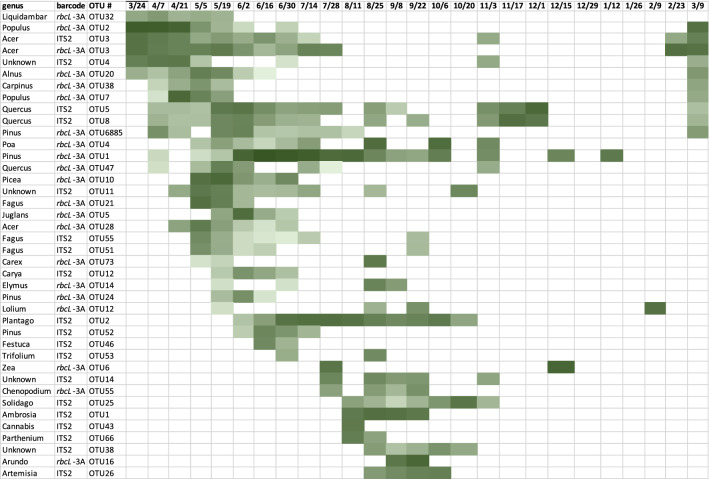
Heat map of the 40 most abundant OTUs found in 14-day dust samples collected in Lexington, MA. The OTU number, barcode used, and assigned genus according to NCBI Genbank are listed, as is the relative abundance in the sample collected on the date indicated. The shade of color indicates the number of reads from a minimum of 10 (light green) to roughly 50,000 (dark green).

Using a relatively high stringency for OTUs (> 2 reads, present in 3 of 3 sequencing replicates, relative abundance > 10^–4^), there was an average of 42.2 ITS2 and 40.4 *rbcL*-3A OTUs per MA dust sample, with a large variance in the OTU number that depended on the date (time of year) of dust accumulation and collection (Fig. 2). There was a significant difference (*p* = 1.6 × 10^–5^) in the number of OTU found in each season. Over 200 total ITS2 and *rbcL*-3A OTUs present in triplicate samples were generated in mid to late spring (April and May 2016). The number of OTUs decreased in samples over the progression from summer to fall and reached a minimum in winter, when samples collected between November 2016 to February 2017 yielded 20 or fewer total OTUs, including two samples with zero OTU. Overall, 2 to 6 times fewer OTUs were recovered from winter samples. In early 2017, the OTU number per sample started to increase with the onset of spring. As expected, there was substantial seasonal variation in the presence of most individual OTUs, with the date of maximum abundance dependent on the taxon. This is illustrated in Fig. 3, which shows the abundance of detected OTUs with the highest read counts according to time of year. There appeared to be three main groups of OTU: those most prevalent in early spring, those most prevalent in late spring/early summer, and those most prevalent in late summer.

Surprisingly, the length of time that slides were exposed to the environment had only a minor impact on the number of OTUs per sample, with no significant difference (*p* = 0.51) in the number of OTUs in dust collected after coincident 14-, 28-, or 56-day exposure (Fig. 2). This indicated that substantial plant material was deposited on a slide in 14 days or less. With few exceptions, the OTU content of coincident 14-, 28-, and 56-day samples was largely similar. The most prevalent OTUs were represented in all (14-, 28-, and 56-day) samples (Supplementary Fig. 1) and any differences were represented by lower abundance OTUs.

The average number of plant OTUs recovered from dust samples from MA was 82.6 ± 73.5, while that from FL, NM, or SC averaged 133.2 ± 105.4, 135.5 ± 72.7, and 132.0 ± 138.1, respectively. Though MA dust samples yielded fewer OTU than those from other collection sites, this difference was not statistically significant (*p* = 0.68). The number of OTUs recovered in samples from NM and SC displayed similar seasonal variation to those from MA, with more recovered in early spring to early summer than in December through February (Supplementary Fig. 2). However, OTUs in samples from FL showed a seasonal variation with a different pattern relative to the other sites, with the most OTU/sample recovered in December through February.

### Estimation of geographic origin

The taxonomy of the plant OTUs derived from DNA extracted from dust were assigned by matching to plant barcode records in NCBI Genbank using a 100% identity threshold. Only 38.3 ± 19.2% of the total OTUs per sample were defined at the species level, the remainder were discarded. 43.6% of the ITS2 OTUs matched a single species and 69.2% matched 5 or fewer species, while 24.7% and 60.3% of the *rbcL*-3A OTUs matched one and less than five species, respectively (Supplementary Fig. 3). This showed that ITS2 performed better that *rbcL*-3A in assigning single plant species using the defined parameters.

**Figure 4:**
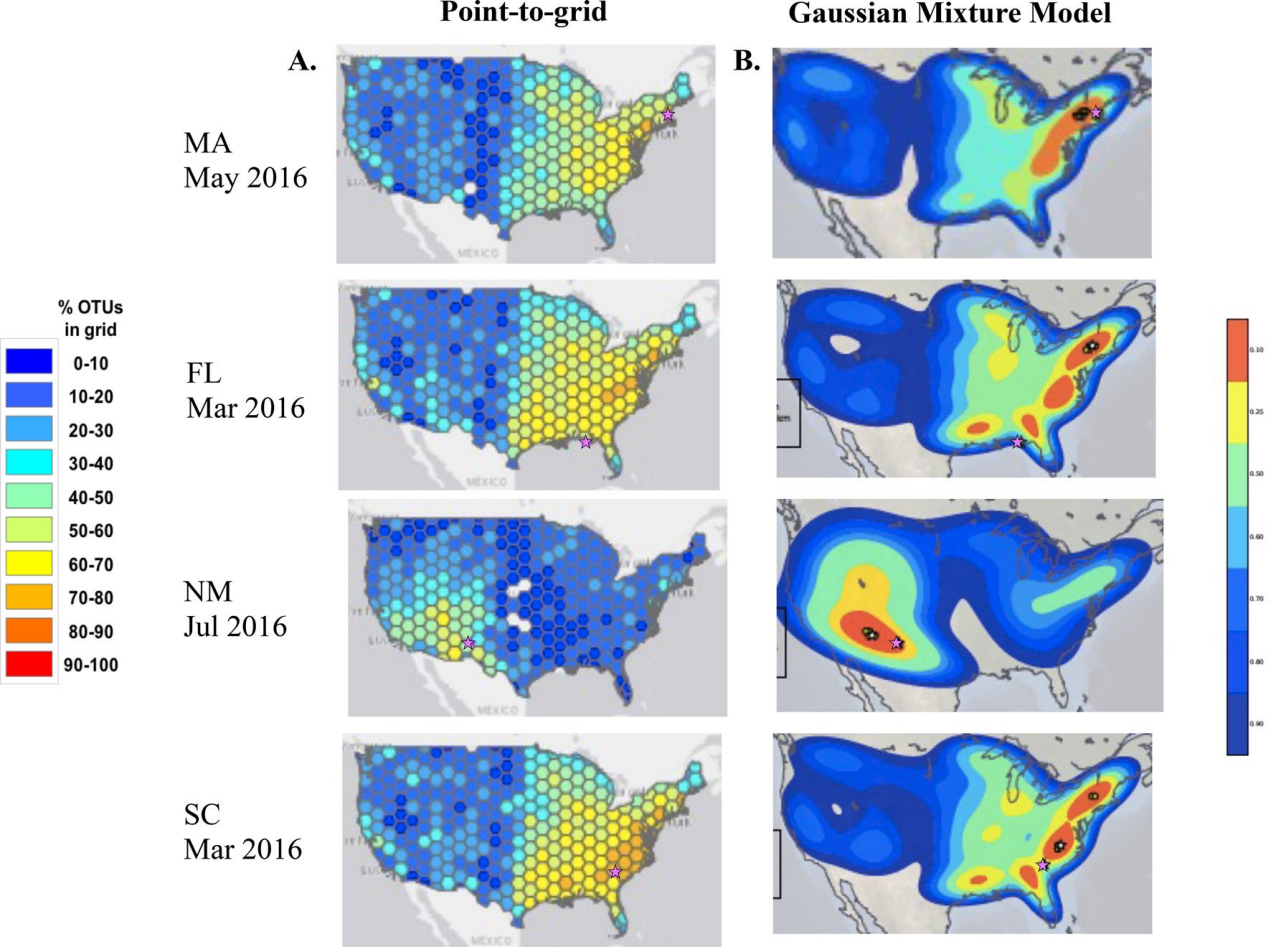
(**A**) Point-to-grid maps displaying overlaid distributions from OTU using ITS2 and *rbcL*-3A minibarcodes generated from 14-day dust samples collected from four locations on the dates indicated. Grid color represents the percentage of total OTU in that sample that had a threshold number of point occurrence records within that grid. The location of sample collection is indicated (pink star). (**B**) Maps showing the result of Gaussian modeling model fitting to the data generated in the point-to-grid map. The location of sample collection is indicated (pink star) as are the locations of the top five peaks used to calculate AT5PE. Maps were generated using python packages interacting with ArcGIS v10.4, as described in “Methods”.

A majority of the point-to-grid maps, which showed the percentage of OTU from that sample that had at least two occurrence records within each 250 km^2^ grid, correctly indicated the region of sample origin within the U.S., meaning that grids with the highest percentage of plant OTUs from that sample co-located within the region of the collection site. Examples of point-to-grid maps generated from each collection site are shown in Fig. 4. Gaussian mixture modeling (GMM) was applied to the point-to-grid maps generated from 14-day samples from the four sites to enable quantitation of accuracy (using the TP) and resolution (using the AT5PE) as described in “Methods” (Fig. 4B). TP indicated the percentage of grids that contain fewer OTU than the grid with the actual sample location and was used with a cutoff of 90%. At that threshold, the grid containing the truth point contained more mapped OTUs than 90% of all grids. AT5PE indicated the mean distance (in km) of the truth site from the five highest probability peaks as determined by our analysis. We utilized an AT5PE of 600 km as a threshold, and considered a positive for regional attribution to hit both thresholds, > 90% TP and < 600 km AT5PE.

**Figure 5:**
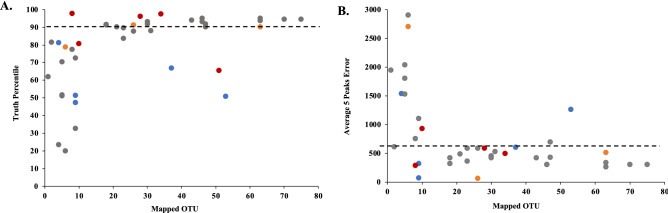
Correlation of total mapped OTU in a sample to its attribution accuracy metrics. Dust samples collected after 14-day exposure in MA (gray), FL (blue), NM (red), or SC (orange) were subject to plant metabarcoding and the resulting OTUs were mapped using our attribution pipeline. (**A**) Relationship between mapped OTU and TP, with a 90% TP cutoff indicated. (**B**) Relationship between mapped OTU and AT5PE, with a 600 km cutoff indicated.

When 14-day dust samples across all four sites were analyzed, the TP was greater than 90% in 20 of 42 (47.6%), the AT5PE was < 600 km in 24 of 42 (57.1%) and 20 of 42 (47.6%) were deemed to produce positive regional geolocation (Table 1). The geolocation accuracy varied notably by collection site, with MA (53.5%), NM (60.0%), and SC (50.0%) showing a similar percentage of positives, while FL samples yielded none. The number of mapped OTUs found in a dust sample appeared to substantially impact the TP and AT5PE (Fig. 5). With both metrics, samples that yielded fewer than 20 mapped OTUs showed greatly reduced attribution accuracy (TP and AT5PE) compared to those with 20 or more OTUs (Table 1). 18 of 42 samples contained fewer than 20 OTUs, 13 of which were collected October through February. When considering only those samples with more than 20 OTUs, 16 of the 24 (66.7%) yielded positive attribution, with a similar variance by the site of sample collection. The duration of dust accumulation, 14, 28, or 56 days, did not have a substantial impact on attribution accuracy and there was a similar threshold of 20 OTUs with samples where dust was collected for more than 14 days (Supplementary Table 1).

**Table 1:** Attribution metrics for 14-day dust samples.

	All samples				Samples with ≥ 20 OTUs			
	#	≥ 90%TP	< 600 km AT5PE	True positive	#	≥ 90% TP	< 600 km AT5PE	True positive
FL	5	0	2	0	2	0	0	0
MA	28	15	17	15	16	13	13	12
NM	5	3	3	3	4	2	2	2
SC	4	2	2	2	2	2	2	2
all sites	42	20	24	20	24	20	17	16

Total number of 14-day dust samples, or the subset of 14-day dust samples with ≥ 20 OTU, that yielded a ≥ 90% TP, less than 600 km AT5PE, or true positive geographic attribution from analysis of constituent plant eDNA from samples collected at the sites indicated. A true positive attribution was defined ≥ 90% TP with < 600 km AT5PE.

Though dependent of the location of dust collection, these data demonstrated that airborne plant DNA in settled dust combined with available species distribution information could accurately estimate the region of origin from a high proportion of samples. With sufficient OTUs, two thirds of the dust samples yielded positive regional attribution. Samples that did not produce correct regional attribution generally had a dispersed OTU distribution, i.e., poor resolution, and did not define an incorrect possible region or origin. These would have been indicated by < 90% TP and 600 km or less AT5PE.

### Dust samples from other U.S. locations

**Figure 6:**
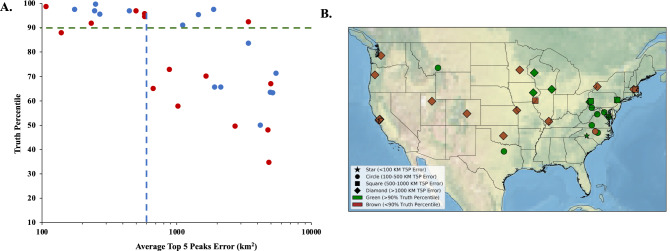
Attribution achieved with plant eDNA derived from 31 WLOH dust samples. (**A**) Correlation of TP to AT5PE in WLOH dust samples in samples with 20 or more (blue) or less than 20 (red) mapped OTUs. Cutoffs for 90% TP and 600 km AT5PE are shown. (**B**) Map of the site of sample collection of the 31 WLOH samples in addition to the attribution accuracy (TP) and resolution (AT5PE) determined from assessment of plant eDNA from dust samples. The color indicates a TP of greater (green) or less than (brown) 90%. The shape of the marker indicates AT5PE of < 100 km (star), 100–500 km (circle), 500–1000 km (square), or > 1000 km (diamond). Maps were generated using python packages interacting with ArcGIS v10.4, as described in “Methods”.

To characterize the achievable geographic attribution accuracy and resolution with dust samples collected from a broader set of locations, metabarcoding was performed on 31 environmental dust samples from different U.S. locations that were collected as part of the Wild Life Our Homes (WLOH) citizen science project^34^. When these samples were analyzed using our pipeline, 10 of the 31 dust samples (32%) generated minibarcodes that resulted in positive attribution (TP > 90%, AT5PE < 600 km) (Fig. 6A). The number of OTUs appeared to have less of an impact on the attribution accuracy with this sample set, and the percentage of positive attribution improved only slightly when samples with more than 20 mapped OTU were considered. Mapping the 31 samples to assess the impact of the region of sample origin on the attribution accuracy and resolution showed that the highest attribution accuracy and precision were achieved with samples from Montana, Texas, and the Middle Atlantic Region. Samples from the west coast of the U.S. and Midwest produced reasonable accuracy (75% or higher truth percentage) but generally low resolution (Fig. 6B). This suggested that OTUs derived from samples from these locations may be less informative, though many more samples would have to be processed to generate significance. It is worth noting that the WLOH sampling method differed from that used for the louvered shelters in that dust accumulation in the WLOH samples was not standardized so that accumulation occurred over a variable duration longer than 14 days. In addition, exposure to environmental factors, which can affect DNA stability, was less controlled.

## Discussion

We have utilized currently available plant sequence and species distribution data to demonstrate a streamlined method for exploiting airborne plant eDNA in dust for forensic attribution. Plant barcodes generated by standard metabarcoding techniques were fed into a data processing pipeline that demonstrated trace airborne plant DNA found in dust can provide an accurate estimate of regional geographic attribution (within 600 km or less) from nearly half of samples collected.

One unique aspect of our pipeline was the use of publicly available biogeographic data from BISON for determination of the geographic distribution of the species found in the samples. Our objective was to determine if currently-available reference data like that in BISON, which has over 400 million species observation records from across the U.S., could be applied to attribution determination to avoid the cost and time needed for sample collection and analysis to create a new reference dataset. For our pipeline, the total area covered by observation records of a plant species was used as an indicator of its geographic distribution, then individual species distributions were normalized and merged into OTU distributions, which were then overlaid using a geographic information system (GIS). This generated a map that indicated the geographic areas with the largest OTU distribution overlap to provide an estimate of the sample origin.

Two different sets of dust samples were used to test using our attribution system. In the first set, consisting of 80 dust samples collected using standardized equipment at four U.S. locations, roughly 40% provided accurate regional attribution estimates. The second set, comprised of 31 samples collected by citizen scientists as part of the WLOH project from different U.S. locations (Supplementary Table 3), yielded 32% with accurate attribution estimates. These percentages increased if samples with less than a threshold of 20 detected OTUs were excluded. A majority (18 of the 29) of the samples that had fewer than 20 OTUs were collected between the months of November and February, which showed that airborne eDNA in dust collected in the winter was less able to support accurate attribution. This confirmed our expectation that the amount of available airborne free plant eDNA or DNA derived from plant-derived particles such as pollen, seeds, and spores is reduced in winter. Snow cover may also have impacted aerosol dispersion of soil-associated plant particles. Notably, there was no evidence of erroneous attribution, where the site of origin was estimated to be in an incorrect regional location, with samples from either set. In samples that did not yield positive attribution, the estimated area of attribution was broad and undefined, meaning there were no samples with a low TP and AT5PE < 600 km^2^.

The data also indicated that 14 days was a sufficient time for dust collection to capture the most prevalent OTU, which implied that an object residing at a location for only 14 days or less (depending on the season) could accumulate an identifiable signature. Further investigation into samples with outdoor environmental exposure shorter than 14 days is needed to determine the minimum amount of time required for attributable signature accumulation, but preliminary studies have indicated a shorter duration will suffice.

As indicated above, the factor most impacting the geographic attribution accuracy and resolution was the number of mapped OTUs (Figs. 4, 5). This is partly due to pipeline methodology, which utilized all mapped OTUs found in a sample but gained the most valuable provenance information from the subset that had narrow geographic distributions. The number of these valuable mapped OTUs increased with the total number of OTUs. OTU number is not only impacted by the amount and variety of plant eDNA deposited on the slide surface, which reflects the local plant abundance/diversity, the collection duration, and season of dust accumulation, but also by physical factors such as air flow through the louvered shelter, exposure to sunlight, and precipitation. Our collection methodology promoted accumulation of fresh airborne material that was protected from UV light and precipitation. Attribution capability would be expected to be reduced using dust samples more exposed to the environment, where the degradation rate of eDNA could be substantially higher^43^. This is consistent with fewer OTUs and lower attribution capability using dust from more exposed environmental surfaces, such as the door top collection location of the the WLOH samples.

The ITS2 and *rbcL*-3A plant minibarcode primer pairs were selected over other primer sets, such as those targeting the chloroplast loci *trnL*, *rbcL*, or *matK* or the nuclear ITS region^18,44–48^, due their OTU yield, taxonomic identification, and representation in NCBI Genbank. Chloroplast barcodes typically amplify from eDNA better than nuclear-based barcodes due to the presence of multiple chloroplast genome copies. However, using our protocol, the primer sets for minibarcodes corresponding to the chloroplast *matK* region produced substantially fewer amplicons than the other primer sets. The *trnL* minibarcode primer set regularly produced the most reads and OTUs when compared directly to other primer sets, consistent with its high amplification efficiency with degraded DNA/eDNA due to its shorter length^21^. However, *trnL* minibarcode sequences were less able to discriminate OTUs at the species level so this primer pair was not as useful as the ITS2 or *rbcL* sets for our pipeline. Two different ITS2 minibarcode primer sets, including the pair used in this study, and *rbcL* minibarcode primer pairs targeting both the 5′ and 3′ regions of the gene amplified most consistently and produced the most mapped OTU (data not shown). The inclusion of additional minibarcode primer sets that delineate plant species unresolved using the ITS2 or *rbcL*-3A minibarcodes would likely improve the number of plant OTUs useful for attribution, though this would increase the chance of having multiple OTUs representing the same species.

The number of mapped OTUs was highly impacted by inefficiencies in matching to the NCBI Genbank and BISON databases. Recent estimates are that barcode sequences from 25 to 40% of the roughly 390,000 plant species in the world^49^ are represented in NCBI Genbank, with the estimated coverage of the 51,000 U.S. plant species higher^50^. However, these estimates include entries representing all plant barcodes, meaning that for any one barcode there are considerable gaps in taxonomic coverage. In addition, the short sequence length of minibarcodes, necessary for compatibility with next generation sequencing, limited their ability to discriminate among plant species records in the NCBI Genbank database. One OTU matched a single Genbank species record 50 to 60% of the time, with the majority of the remainder matching 2 to 10 species (Supplementary Fig. 3). In fact, we used a similarity threshold of 100% because a lower threshold increased the number of OTUs assigned to the same species while not substantially increasing the number of new species identified. Recently developed sequence alignment algorithms that are alternatives to nBLAST may enable improved plant taxonomic assignment using minibarcodes^51^, as may use of longer barcode sequences generated through the use of improved sequence chemistry, amplification and sequencing of long barcode amplicons using nanopore-type sequencing, chloroplast genome sequencing, or genome skimming^30,52^.

The quality and availability of plant biogeographic reference data was perhaps the most important factor affecting attribution applicability, accuracy, and resolution. BISON provided a tremendous wealth of information for determining species distributions to enable a proof-of-concept demonstration, but limitations in taxonomic, geographic, and temporal coverage^53^ impacted the achievable attribution resolution and accuracy of our pipeline. Incomplete taxonomic coverage, exacerbated by the difficulty in harmonizing the different taxonomic nomenclature systems used by NCBI Genbank and BISON, affected the ability to fully characterize the biodiversity distributions of sample OTUs. Geographic coverage, or how well the actual distribution of a species is documented by the occurrence records, and uneven spatial distribution of occurrence records also likely impacted the estimation of attribution accuracy^54^. To mitigate these issues, BISON occurrence records can be augmented with data from other available sources, or with species distribution modeling, to enhance both taxonomic and geographic coverage. Lastly, though BISON data is highly curated, there are possible data quality issues due to incorrect taxonomic assignment or duplicate or erroneous entries. The same is true of NCBI Genbank, which is known to have sequence and taxonomic inaccuracies. Curation of these reference datasets could provide a substantial improvement in attribution accuracy.

It should be noted that this study relied heavily on contributions from citizen-scientists, both for the entry of plant distribution data into the BISON database and for the collection of analyzed dust samples as part of the WLOH project. This method of data/sample collection, while having more quality control issues than sceintist-driven collections, provided substantial value to this project. This type of resource could be utilized more often for more efficient and inexpensive environmental data and sample collection, particularly when geographic diversity and coverage is needed.

In summary, this analysis demonstrated that airborne plant eDNA in dust is capable of reproducibly defining the U.S. region of sample origin within a radius of 600 km or less from a sizeable percentage of samples. The capability to acquire a regional estimate of provenance in many trace samples rapidly, without specialized expertise, can provide value to many types of forensic investigation. We believe that, by streamlining metabarcoding protocols (using multiplexing, for instance) and automating data analysis, attribution information could be gained from hundreds of dust samples in days. This could enable rapid screening of the multiple found objects, such as clothing, vehicles, or instruments, to estimate their point or origin or previous location.

## Methods

### Dust collection

**Figure 7:**
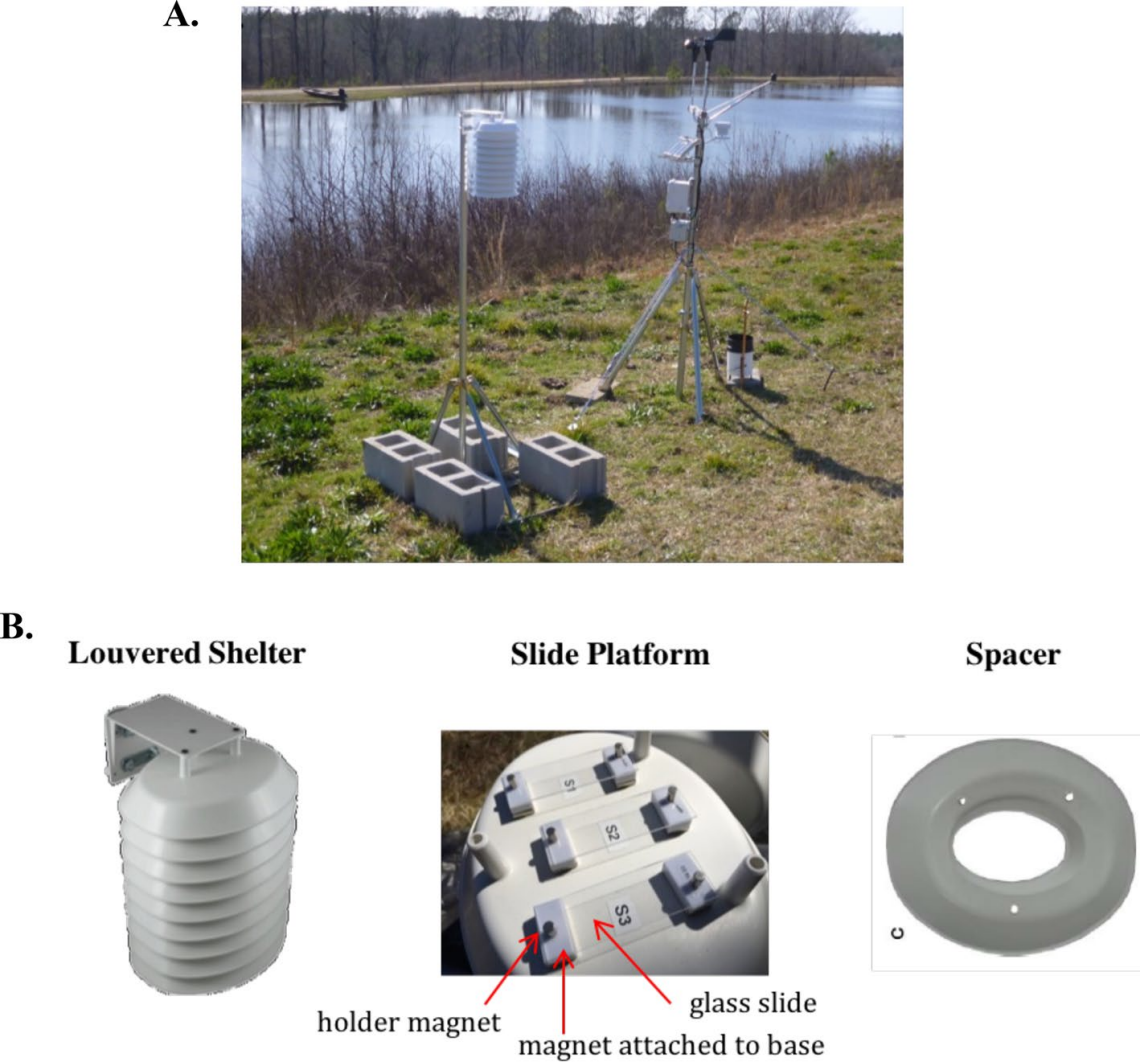
Settled airborne dust collection station. (**A**) Photo from the South Carolina dust collection site of EZ-NPP tripod secured with cinder blocks holding an assembled Ambient Weather SRS100LX radiation shield (louvered shelter). Also pictured is an Onset HOBO U30-RNC Weather Station. (**B**) Diagram of a louvered shelter. A slide platform was created by mounting 6 magnets spaced appropriately to serve as slide holders into a base (center). Clean slides were secured into the holders with additional small magnets. Each complete louvered shelter (left) consisted of 3 sets of a slide platform underneath two spacers (right) added for additional air flow.

Dust was collected on standard 72 × 25 x 1 mm glass microscope slides (e.g., VWR VistaVision 16004-422) that were cleaned with glass cleaner, twice rinsed with distilled water, and dried with compressed air. Nine slides were secured with magnets onto three platforms of a louvered shelter (SRS100LX radiation shield, Ambient Weather, Chandler, AZ, USA) mounted on a tripod one meter off the ground and at least 10 m from buildings or other structures that could impede airflow (Fig. 7). The louvered shelter enabled particle settling, passive collection, on the slides while protecting them from precipitation. Dust was collected on slides at four U.S. locations, Lexington, MA, Panama City, FL, Socorro, NM, and Edgewood, SC, between March 2016 to June 2017. At the FL, NM, and SC sites, which required travel for sampling, dust was collected from slides left undisturbed for 14 days once per season for 12–15 months. This resulted in 4 or 5 slide sets per site. The MA location was located close to our facility so that additional slide sets could be collected: after 14, 28, or 56 days of concurrent environmental exposure. At the MA site, 49 sets of slides were collected: 28 after 14 days, 14 after 28 days, and 7 after 56 days. After environmental exposure, slides were removed from the collection rig and stored at 4 °C within 5 days of collection. Settled particles, comprised of pollen, spores, plant fragments, free DNA, and inorganic material, were gathered from two slides from each set with a single Puritan DNA-free PurFlock Ultra Tipped Applicator with Transport Tubes (Puritan #253306UTTFDNA) wetted with isopropanol. Negative control samples were gathered using the same method from two glass slides that were kept in a closed container after cleaning. After sample collection, swabs were air dried and stored at 4 °C until further processing.

### Wild life our homes samples

In addition to passively collected samples, 31 environmental dust samples from different U.S. locations (Supplementary Table 3) that were collected as part of the Wild Life Our Homes (WLOH) citizen science project^34^ were analyzed. These were a portion of a larger set of paired indoor and outdoor dust samples collected by enrolled citizen scientists between March 2012 and May 2013. Dust was collected by swabbing the upper door trim on an interior door in the main living area of a resident’s home or the upper door trim on the outside surface of an exterior door. Extracted DNA from these 459 samples was initially used in a separate study^55^.

### Metabarcoding and sequencing

DNA was extracted from swabs using the MoBio/Qiagen PowerSoil htp-96 Well Isolation Kit (catalog #12955-4) according to the manufacturer’s recommended protocol. Plant-specific minibarcodes targeting the ITS2 and *rbcL* regions (Supplementary Table 2) were used for their compatibility with next-generation sequencing (NGS) technology and for improved amplification of eDNA. Two minibarcodes were utilized to improve the number of plant species detected, and the OTU obtained with each primer set were combined and analyzed as a group. Minibarcodes were amplified and sequenced by a commercial vendor (Jonah Ventures, LLC, Boulder, CO) using primers with appended 5’ adapter sequences. Each 25 μl amplification reaction consisted of 12.5 μl GoTaq PCR mastermix (Promega cat # M5133), 0.5 μl each forward and reverse primer (0.2 μM final concentration), 1 μl extracted eDNA or extraction buffer (blank), and 10.5 μl DNase/RNase-free water. DNA was PCR amplified by denaturation at 94 °C for 5 min, followed by 40 cycles of 30 s at 94 °C, 30 s at 56 °C, and 45 s at 72 °C, and a final elongation at 72 °C for 10 min. PCR amplifications were confirmed using agarose gel electrophoresis. Amplicons were then purified using MoBio UltraClean-htp 96 well PCR Clean-Up Kit (catalog # 12596-4) and each combination of sample and primer pair was assigned its own sequencing barcode. Pools were sequenced in triplicate using an Illumina MiSeq using the Reagent Kit v2 300-cycle kit (catalog # MS-102-2002). Sequences were demultiplexed using Golay barcodes^56^ via QIIME v1.9.1^57^ and merging of paired end reads and trimming were performed with USEARCH^58^. CUTADAPT v1.8.1 was then used to identify and remove remaining primer and adapter regions^59^. Sequences were quality trimmed to have a maximum expected number of errors per read of less than 0.5. General quality filtering and OTU construction was completed as per the UPARSE pipeline^60^ with de novo clustering at 99% sequence similarity. These parameters help to ensure that individual reads are correctly mapped to their respective OTU. Merged reads from ITS2 and single reads from *rbcL*-3A were clustered into OTUs (99% similarity using a de novo method). OTUs that had fewer than 3 reads, those that were not present in 3 of the 3 sequencing replicates, or those that had a relative abundance less than 10^–4^ were culled. This eliminated most of the OTUs (60.9% to 92.4%) from each sample, particularly those of lower abundance. Any OTUs present in the negative control (swab of clean slide) and PCR blank (no template DNA) were eliminated using this stringency filtering. Statistical analysis on the number of OTUs was performed using analysis of variance (ANOVA).

### Taxonomic assignment and species distribution determination

Taxa were assigned using the Genbank nBLAST homology inquiry tool using a query threshold of 100%. 40 to 50% of OTUs matched more than one species record in Genbank using a 100% homology threshold (Supplemental Fig. 3). These species were from the same genera, for instance *Pinus*, where the ITS2 or *rbcL*-3A minibarcode sequence did not discriminate certain species. In this case, all matching species were retained for subsequent analysis. However, an individual plant species was only represented once per sample, even if it matched more than one OTU. Genbank taxonomic nomenclature was not completely consistent with that of BISON, so assigned taxa were edited by trimming to only species, i.e. removing subspecies and variety names, then processed using the R package taxize^61,62^ to better align species names to those present in BISON. As a final step, manual curation corrected misspellings and removed unassigned taxa (uncultured, environmental sample, etc.). OTU taxonomy was displayed at the genus level in Figs. 3 and Supplementary Fig. 1 for better visualization.

Occurrence data was retrieved from the BISON database by using the taxonomic serial number (TSN), since ~ 98% of entries in BISON had an associated TSN, then by genus and species. Record retrieval from the BISON database was initially performed using a custom R script that retrieved records from the BISON application program interface (API) using the rbison package^63^. The records retrieved were modified to exclude groups based on certain data fields, for instance records that have been flagged for having apparently invalid or mismatched latitude/longitude coordinates, countries, or continents. Up to 10^5^ species occurrence records were retrieved for a single query. Some species produced no recorded occurrences with a geographic reference due to the lack of complete taxonomic coverage of the BISON biogeography database and the inability to resolve all nomenclature inconsistencies.

### Mapping

The ArcGIS v10.4 geographic information system (GIS) package (Environmental Systems Research Institute (ESRI), Redlands, CA, https://desktop.arcgis.com/en/system-requirements/10.4/arcgis-desktop-system-requirements.htm) was used to estimate the geographic attribution achieved from the species distributions associated with each sample. The primary output was a point-to-grid U.S. map that, within each grid, displayed the percentage of OTU from a sample with 2 or more occurrence records in that grid. To do this, the total observation records for each species assigned to an OTU were merged to generate an OTU-based geographic distribution. This step improved the attribution accuracy of the pipeline compared to consideration of each species within a sample independently. OTU-based geographic distributions were converted to an analytical layer that was intersected with a global 250-km grid map displaying the number of occurrences per grid. Species prevalence in each grid was normalized by positive (two or more occurrences) or negative (less than two occurrences) designation. The maps of the normalized OTU-based geographic distributions for every OTU in a sample were overlaid and, for each grid, the proportion of the total OTU was calculated. This method minimized the impact of OTUs with wide geographic distributions (with occurrence records in many grids) that were less informative for geographic attribution, and enabled detection of OTU with a more localized distribution, which were more informative for geographic attribution. These steps were merged into a custom Python script, that utilized the ArcGIS v10.4 library Arcpy (2014, ESRI) to enable automated analysis of multiple data sets and high throughput mapping. A “basemap” python package^64^ (Python Matplotlib BaseMap v1.1: https://matplotlib.org/basemap/) was used to build maps, the “scikit-learn” python package for the Gaussian mixture model and metrics, the “arcpy” python module as well as numpy and pandas^65^ for interacting with ArcGIS v10.4 processes.

To better compare point-to-grid maps, geographic attribution metrics were generated from a Gaussian Mixture Model (GMM) fitted to the point-to-grid map (Fig. 4). The GMM was fitted with Scikit-learn using the variational inference extension to the expectation maximization (EM) algorithm with the Dirichlet process to determine the number of OTUs in the mixture^66^. This incorporated the analytical advantages of having a probabilistic model for the PtG data while retaining the robustness to low quality data (normalization) provided by the PtG method. The primary metrics utilized truth data, in this case the actual collection location, to determine the accuracy and resolution of the geographic attribution estimated from the plant OTU. Accuracy, designated by truth percentage (TP), indicated how closely an attribution map came to measuring the location of sample origin by measuring the likelihood percentile of the truth point in the data, i.e., the percentage of grids with less than the OTU count value in the grid containing the location of sample collection. A higher TP reflected better accuracy. Attribution resolution described the spatial precision of the data, with the primary metric calculated by determining the distance(s) between the truth point (location of the sample origin) and top 5 points (map grids) with highest likelihood/OTU number. This was referred to as the average top 5 peaks error (AT5PE), and was the standard indicator of attribution resolution.

## Supplementary Information

Supplementary Information.

## Data Availability

The datasets used and/or analyzed during the current study are available from the corresponding author on reasonable request.

## References

[1] Stoney D, Bowen A, Stoney P (2013). Inferential source attribution from dust: Review and analysis. Forensic Sci. Rev..

[2] Locard E (1930). The analysis of dust traces. Am. J. Police Sci..

[3] Wickenheiser RA (2002). Trace DNA: A review, discussion of theory, and application of the transfer of trace quantities of DNA through skin contact. J. Forensic Sci..

[4] Adams-Groom B (2015). Frequency and abundance of pollen taxa in crime case samples from the United Kingdom. Grana.

[5] Bryant VM, Jones GD (2006). Forensic palynology: Current status of a rarely used technique in the United States of America. Forensic Sci. Int..

[6] Laurence AR, Bryant VM (2019). Forensic palynology and the search for geolocation: Factors for analysis and the Baby Doe case. Forensic Sci. Int..

[7] Mildenhall D, Wiltshire PE, Bryant VM (2006). Forensic Palynology: Why Do It and How It Works.

[8] Taylor B, Skene K (2003). Forensic palynology: Spatial and temporal considerations of spora deposition in forensic investigations. Aust. J. Forensic Sci..

[9] Halbritter H, Ulrich S, Grímsson F, Weber M, Zetter R, Hesse M (2018). Methods in Palynology.

[10] Stillman E, Flenley JR (1996). The needs and prospects for automation in palynology. Quatern. Sci. Rev..

[11] Walsh KA, Horrocks M (2008). Palynology: Its position in the field of forensic science. J. Forensic Sci..

[12] Christou, C., Jacyna, G., Goodman, F., Deanto, D., Masters, D. (eds.) Geolocation analysis using Maxent and plant sample data. In *2015 IEEE International Symposium on Technologies for Homeland Security (HST)*; 2015: IEEE.

[13] Goodman, F., Doughty, J., Gary, C., Christou, C., Hu, B., Hultman, E., et al. (eds.) PIGLT: A pollen identification and geolocation system for forensic applications. In *2015 IEEE International Symposium on Technologies for Homeland Security (HST)*; 2015: IEEE.

[14] Hebert PD, Gregory TR (2005). The promise of DNA barcoding for taxonomy. Syst. Biol..

[15] Kress WJ (2017). Plant DNA barcodes: Applications today and in the future. J. Syst. Evol..

[16] Tautz D, Arctander P, Minelli A, Thomas RH, Vogler AP (2003). A plea for DNA taxonomy. Trends Ecol. Evol..

[17] Bell KL, Burgess KS, Okamoto KC, Aranda R, Brosi BJ (2016). Review and future prospects for DNA barcoding methods in forensic palynology. Forensic Sci. Int. Genet..

[18] Chen S, Yao H, Han J, Liu C, Song J, Shi L (2010). Validation of the ITS2 region as a novel DNA barcode for identifying medicinal plant species. PloS one..

[19] Fahner NA, Shokralla S, Baird DJ, Hajibabaei M (2016). Large-scale monitoring of plants through environmental DNA metabarcoding of soil: Recovery, resolution, and annotation of four DNA markers. PloS One..

[20] Hollingsworth PM, Forrest LL, Spouge JL, Hajibabaei M, Ratnasingham S, Group CPW (2009). A DNA barcode for land plants. Proc. Natl. Acad. Sci..

[21] Taberlet P, Coissac E, Pompanon F, Gielly L, Miquel C, Valentini A (2006). Power and limitations of the chloroplast trn L (UAA) intron for plant DNA barcoding. Nucleic Acids Res..

[22] Kraaijeveld K, De Weger LA, Ventayol García M, Buermans H, Frank J, Hiemstra PS (2015). Efficient and sensitive identification and quantification of airborne pollen using next-generation DNA sequencing. Mol. Ecol. Resour..

[23] Müller-Germann I, Vogel B, Vogel H, Pauling A, Fröhlich-Nowoisky J, Pöschl U (2015). Quantitative DNA analyses for airborne birch pollen. PloS One.

[24] Matsuki Y, Isagi Y, Suyama Y (2007). The determination of multiple microsatellite genotypes and DNA sequences from a single pollen grain. Mol. Ecol. Notes.

[25] Bell KL, Fowler J, Burgess KS, Dobbs EK, Gruenewald D, Lawley B (2017). Applying pollen DNA metabarcoding to the study of plant–pollinator interactions. Appl. Plant Sci..

[26] Keller A, Danner N, Grimmer G, Ankenbrand M, Von Der Ohe K, Von Der Ohe W (2015). Evaluating multiplexed next-generation sequencing as a method in palynology for mixed pollen samples. Plant Biol..

[27] Sickel W, Ankenbrand MJ, Grimmer G, Holzschuh A, Härtel S, Lanzen J (2015). Increased efficiency in identifying mixed pollen samples by meta-barcoding with a dual-indexing approach. BMC Ecol..

[28] Prosser SW, Hebert PD (2017). Rapid identification of the botanical and entomological sources of honey using DNA metabarcoding. Food Chem..

[29] Baksay S, Pornon A, Burrus M, Mariette J, Andalo C, Escaravage N (2020). Experimental quantification of pollen with DNA metabarcoding using ITS1 and trnL. Sci. Rep..

[30] Bell KL, de Vere N, Keller A, Richardson RT, Gous A, Burgess KS (2016). Pollen DNA barcoding: current applications and future prospects. Genome.

[31] Ruppert KM, Kline RJ, Rahman MS (2019). Past, present, and future perspectives of environmental DNA (eDNA) metabarcoding: A systematic review in methods, monitoring, and applications of global eDNA. Global Ecol. Conserv..

[32] Giampaoli S, Berti A, Di Maggio R, Pilli E, Valentini A, Valeriani F (2014). The environmental biological signature: NGS profiling for forensic comparison of soils. Forensic Sci. Int..

[33] Young J, Austin J, Weyrich L (2017). Soil DNA metabarcoding and high-throughput sequencing as a forensic tool: considerations, potential limitations and recommendations. FEMS Microbiol. Ecol..

[34] Barberán A, Ladau J, Leff JW, Pollard KS, Menninger HL, Dunn RR (2015). Continental-scale distributions of dust-associated bacteria and fungi. Proc. Natl. Acad. Sci..

[35] Grantham NS, Reich BJ, Pacifici K, Laber EB, Menninger HL, Henley JB (2015). Fungi identify the geographic origin of dust samples. PLoS One..

[36] Allwood JS, Fierer N, Dunn RR, Breen M, Reich BJ, Laber EB (2020). Use of standardized bioinformatics for the analysis of fungal DNA signatures applied to sample provenance. Forensic Sci. Int..

[37] Grantham NS, Reich BJ, Laber EB, Pacifici K, Dunn RR, Fierer N (2020). Global forensic geolocation with deep neural networks. J. Roy. Stat. Soc. Ser. C (Appl. Stat.).

[38] Damaso N, Mendel J, Mendoza M, von Wettberg EJ, Narasimhan G, Mills D (2018). Bioinformatics approach to assess the biogeographical patterns of soil communities: The utility for soil provenance. J. Forensic Sci..

[39] Lenehan CE, Tobe SS, Smith RJ, Popelka-Filcoff RS (2017). Microbial composition analyses by 16S rRNA sequencing: A proof of concept approach to provenance determination of archaeological ochre. PloS One..

[40] Badgley AJ, Jesmok EM, Foran DR (2018). Time radically alters ex situ evidentiary soil 16S bacterial profiles produced via next-generation sequencing. J. Forensic Sci..

[41] Fierer N (2017). Embracing the unknown: Disentangling the complexities of the soil microbiome. Nat. Rev. Microbiol..

[42] Johnson MD, Cox RD, Barnes MA (2019). The detection of a non-anemophilous plant species using airborne eDNA. PloS One..

[43] Thomsen PF, Willerslev E (2015). Environmental DNA—An emerging tool in conservation for monitoring past and present biodiversity. Biol. Cons..

[44] Dong W, Liu H, Xu C, Zuo Y, Chen Z, Zhou S (2014). A chloroplast genomic strategy for designing taxon specific DNA mini-barcodes: A case study on ginsengs. BMC Genet..

[45] Dunning LT, Savolainen V (2010). Broad-scale amplification of matK for DNA barcoding plants, a technical note. Bot. J. Linn. Soc..

[46] Han J, Zhu Y, Chen X, Liao B, Yao H, Song J (2013). The short ITS2 sequence serves as an efficient taxonomic sequence tag in comparison with the full-length ITS. BioMed Res. Int..

[47] Little DP (2014). A DNA mini-barcode for land plants. Mol. Ecol. Resour..

[48] Meusnier I, Singer GA, Landry J-F, Hickey DA, Hebert PD, Hajibabaei M (2008). A universal DNA mini-barcode for biodiversity analysis. BMC Genomics.

[49] Kew, R. B. G. The state of the world’s plants report–2016. Royal Botanic Gardens, Kew. 2016.

[50] Ulloa CU, Acevedo-Rodríguez P, Beck S, Belgrano MJ, Bernal R, Berry PE (2017). An integrated assessment of the vascular plant species of the Americas. Science.

[51] Li, H., Bai, H., Yu, S., Han, M. & Ning, K. Holmes-ITS2: Consolidated ITS2 resources and search engines for plant DNA-based marker analyses. bioRxiv. 2018:263541.

[52] Coissac E, Hollingsworth PM, Lavergne S, Taberlet P (2016). From barcodes to genomes: Extending the concept of DNA barcoding. Mol. Ecol..

[53] Meyer C, Weigelt P, Kreft H (2016). Multidimensional biases, gaps and uncertainties in global plant occurrence information. Ecol. Lett..

[54] Meyer C, Kreft H, Guralnick R, Jetz W (2015). Global priorities for an effective information basis of biodiversity distributions. Nat. Commun..

[55] Craine JM, Barberán A, Lynch RC, Menninger HL, Dunn RR, Fierer N (2017). Molecular analysis of environmental plant DNA in house dust across the United States. Aerobiologia.

[56] Caporaso JG, Lauber CL, Walters WA, Berg-Lyons D, Huntley J, Fierer N (2012). Ultra-high-throughput microbial community analysis on the Illumina HiSeq and MiSeq platforms. ISME J..

[57] Caporaso JG, Kuczynski J, Stombaugh J, Bittinger K, Bushman FD, Costello EK (2010). QIIME allows analysis of high-throughput community sequencing data. Nat. Methods.

[58] Edgar RC (2010). Search and clustering orders of magnitude faster than BLAST. Bioinformatics.

[59] Martin M (2011). Cutadapt removes adapter sequences from high-throughput sequencing reads. EMBnet J..

[60] Edgar RC (2013). UPARSE: highly accurate OTU sequences from microbial amplicon reads. Nat. Methods.

[61] Chamberlain, S., *et al.* Taxize: taxonomic information from around the web. Version 0.7. 8. 2016.

[62] Chamberlain, S. A. & Szöcs, E. Taxize: Taxonomic search and retrieval in R. F1000Research. 2013;2. 10.12688/f1000research.2-191.v1 PMC3901538 24555091

[63] Chamberlain, S. A. & Boettiger, C. R Python, and Ruby clients for GBIF species occurrence data. PeerJ Preprints; 2017. Report No.: 2167–9843.

[64] Hunter JD (2007). Matplotlib: A 2D graphics environment. Comput. Sci. Eng..

[65] McKinney, W. Python for data analysis: Data wrangling with Pandas, NumPy, and IPython: O'Reilly Media, Inc.; 2012.

[66] Pedregosa F (2011). Scikit-learn: Machine learning in Python. J. Mach. Learn. Res..

