# Supplementary material for: Geographic source estimation using airborne plant environmental DNA in dust
Source: Sci Rep. 2021 Aug 10;11:16238. doi: 10.1038/s41598-021-95702-3 (PMC8355115; doi:10.1038/s41598-021-95702-3)
Supplement: Supplementary file 1 — Supplementary Information. [file 41598_2021_95702_MOESM1_ESM.pdf]

| | | | | 2016 | | | | | | | | | | | | | | | | | | | | | 2017 | | | | | | |
|---|---|---|---|---|---|---|---|---|---|---|---|---|---|---|---|---|---|---|---|---|---|---|---|---|---|---|---|---|---|---|---|
| Barcode | OTU | Genus | Exposure (d) | 3/24 | 4/7 | 4/21 | 5/5 | 5/19 | 6/2 | 6/16 | 6/30 | 7/14 | 7/28 | 8/11 | 8/25 | 9/8 | 9/22 | 10/6 | 10/20 | 11/3 | 11/17 | 12/1 | 12/15 | 12/29 | 1/12 | 1/26 | 2/9 | 2/23 | 3/9 | 3/23 | 4/6 |
| *rbcL* -3A | OTU10 | Picea | 14 | | | | 5331 | 6779 | 543 | 107 | 1369 | | | | | | | | | | | | | | | | | | | | |
| | | | 28 | | | 2503 | | 2801 | | 263 | | | | | | | | | | | | | | | | | | | | | |
| | | | 56 | 559 | | | | 723 | | | | | | | | | | | | | | | | | | | | | | | |
| *rbcL* -3A | OTU13 | Myrica | 14 | | 488 | 197 | 657 | 117 | 95 | | | | | | | | | | | | | | | | | | | | 1339 | | |
| | | | 28 | 486 | | 65 | | 309 | | 5 | | | | | | | | | | | | | | | | | | 1024 | | | |
| | | | 56 | 261 | | | | | | | | | | | | | | | | | | | | | | | | | | | |
| ITS2 | OTU2 | Plantago | 14 | | | | | | 24 | 194 | 3983 | 2705 | 3723 | 4999 | 2111 | 936 | 1994 | 2793 | 252 | | | | | | | | | | | | |
| | | | 28 | | | 5 | | | | 1050 | | 2497 | | 1302 | | 924 | | 1265 | | | | | | | | | | | | | |
| | | | 56 | | | | | 144 | | | | 2280 | | | | 938 | | | | | | | | | | | | | | | |
| ITS2 | OTU26 | Artemisia | 14 | | | | | | | | | | | | 398 | 854 | 1963 | 2400 | | | | | | | | | | | | | |
| | | | 28 | | | | | | | | | | | 494 | | 2692 | | 30 | | 256 | | | | | | | | | | | |
| | | | 56 | | | | | | | | | 108 | | | | 1458 | | | | | | | | | | | | | | | |
| *rbcL* -3A | OTU3 | Acer | 14 | 5344 | 2325 | 1131 | 2030 | 2558 | 731 | 39 | 409 | 678 | 460 | | | | | | | | | | | | | | | 2304 | 6291 | 1777 | 6514 |
| | | | 28 | 2488 | | 1032 | | 1485 | | 78 | | 1627 | | | | | | 3190 | | 9 | | | | | | | | 9881 | | 4797 | |
| | | | 56 | 646 | | | | 786 | | | | 1297 | | | | 2325 | | | | | | | | 3112 | | | | 2937 | | | |
| ITS2 | OTU4 | unknown | 14 | 3108 | 2322 | 2378 | 70 | | | | 15 | | | | | | | | | 151 | | | | | | | | | 320 | | 5223 |
| | | | 28 | 4442 | | | | 1001 | | | | | | | | 25 | | 164 | | 111 | | 0 | | 0 | | 0 | | 402 | | 3016 | |
| | | | 56 | 876 | | | | | | | | | | | | | | | | 253 | | | | 0 | | | | 2198 | | | |
| ITS2 | OTU5 | Quercus | 14 | | 57 | 44 | 83 | 2177 | 3385 | 610 | 333 | 204 | 408 | | 398 | 19 | | | | 993 | 3291 | 10479 | | | | | | | 85 | | 29 |
| | | | 28 | 58 | | 421 | | 71 | | 283 | | 84 | | | | 73 | | | | 653 | | | | | | | | | | 161 | |
| | | | 56 | 44 | | | | 5343 | | | | 1250 | | | | 416 | | | | 186 | | | | | | | | | | | |
| *rbcL* -3A | OTU7 | Populus | 14 | | 9 | 4934 | 2368 | 357 | | | | | | | | | | | | | | | | | | | | | 460 | | |
| | | | 28 | | | 101 | | 3350 | | | | | | | | | | | | | | | | | | | | | | | |
| | | | 56 | 1260 | | | | | | | | | | | | | | | | | | | | | | | | | | | |

**Supplementary Figure 1**

Read abundance of eight example plant OTUs derived from metabarcoding dust samples collected from slides after 14, 28, or 56 days of environmental exposure from the Lexington, MA site on the dates indicated. Numbers and color intensity represent the reads assigned to the OTU generated with either the ITS2 or *rbcL*-3A primer set (barcode).

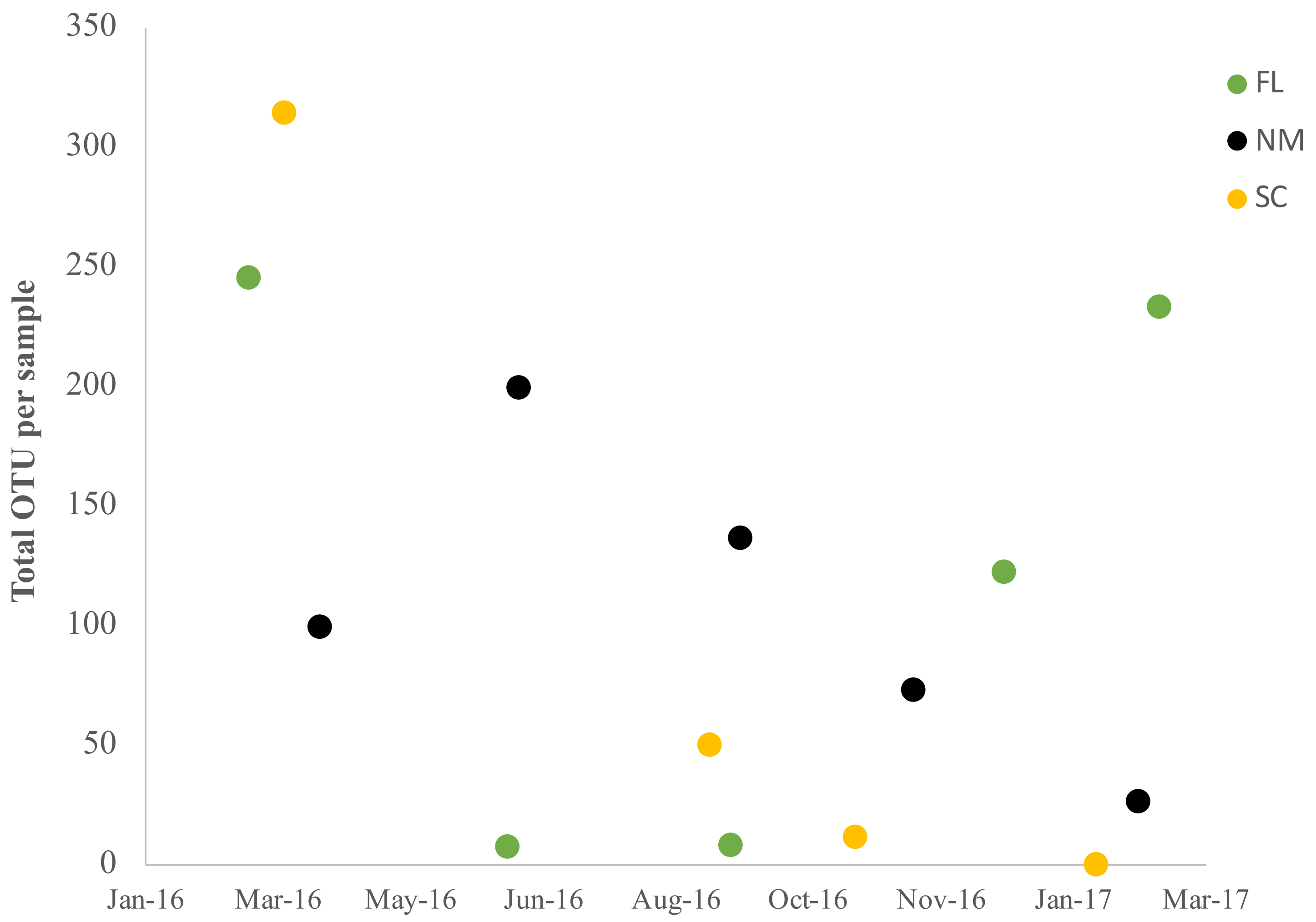


**Supplementary Figure 2**

Seasonal variation in the number of OTU recovered from dust samples collected from Panama City, FL (green), Socorro, New Mexico (black), or Edgefield, South Carolina (yellow) on the date indicated. Dust accumulated for 14 days on each sample.

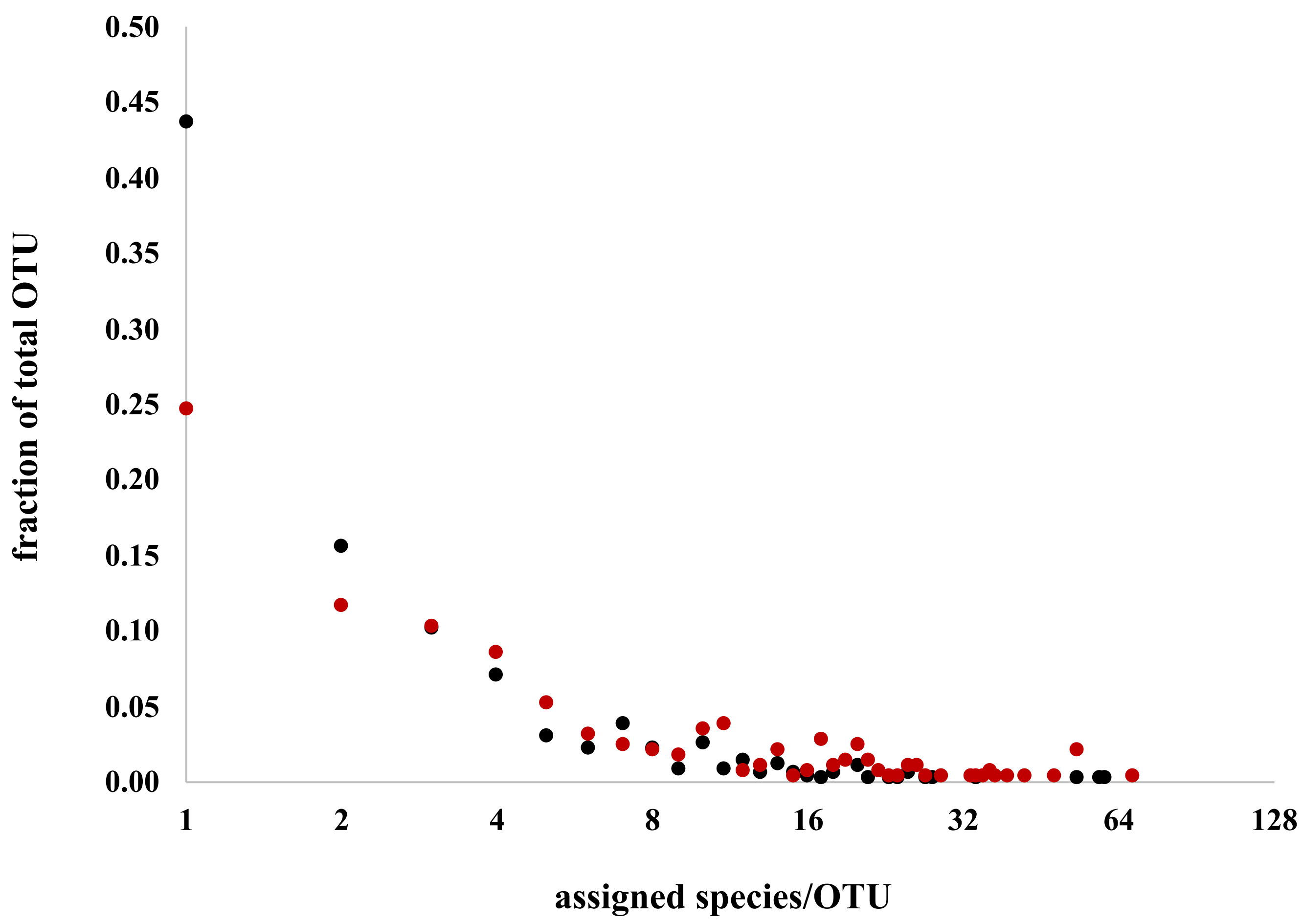


**Supplementary Figure 3**

Fraction of OTU generated by the ITS2 (black) or *rbcL*-3A (red) minibarcodes assigned to one or more species after 100% similarity matching to barcode sequences in NCBI Genbank.

**Supplementary Table 1**

Number of total dust samples (all collection durations), or the subset of those with ≥ 20 OTU, that yielded a ≥ 90% TP, less than 600 km AT5PE, or true positive geographic attribution from analysis of constituent plant eDNA from samples collected at the sites indicated. A true positive attribution is defined ≥ 90% TP with < 600 km AT5PE.

| | all samples | | | | samples with 20+ OTU | | | |
|---|---|---|---|---|---|---|---|---|
| | # | ≥ 90% TP | < 600 km AT5PE | True positive | # | ≥ 90% TP | < 600 km AT5PE | True positive |
| **FL** | 10 | 1 | 3 | 0 | 5 | 0 | 0 | 0 |
| **MA** | 48 | 27 | 31 | 24 | 32 | 23 | 28 | 27 |
| **NM** | 10 | 6 | 5 | 5 | 7 | 6 | 5 | 5 |
| **SC** | 10 | 5 | 4 | 3 | 7 | 5 | 5 | 4 |
| **all sites** | 78 | 39 | 43 | 32 | 51 | 34 | 38 | 36 |

**Supplementary Table 2**

Description of ITS2 and *rbcL* minibarcode primers used in this study.

| barcode locus | | primer | sequence | reference |
|---|---|---|---|---|
| ITS2 | F | ITS2 S2F Plant | ATGCGATACTTGGTGTGAAT | Chen et al., 2010 |
| | R | ITS4 | TCCTCCGCTTATTGATATGC | White, 1990 |
| *rbcL* 3' region 370-507 | F | rbcLF2 | TGTTTACTTCCATTGTGGGTAATG | Murphy et al., 2011; Little, 2014 |
| | R | rbcLR3a | TTCGGTTTAATAGTACAGCCCAAT | |

**Supplementary Table 3**

Locations of WLOH dust sample collection.

| City | State | Type |
|---|---|---|
| Maple Valley | WA | One-family house attached |
| Pleasant Prairie | WI | One-family house detached |
| Berkeley | CA | One-family house detached |
| Rocky Mount | VA | One-family house detached |
| Timberville | VA | One-family house detached |
| Corvallis | OR | Duplex or two-family house |
| Raleigh | NC | One-family house detached |
| Livingston | MT | One-family house detached |
| Colorado Springs | CO | One-family house detached |
| Salt Lake City | UT | One-family house detached |
| Chippewa Falls | WI | One-family house detached |
| Fort Worth | TX | Low rise apartment |
| Cedar Rapids | IA | One-family house detached |
| South Haven | MN | One-family house detached |
| Moore | OK | One-family house detached |
| Charlotte | NC | One-family house detached |
| Littleton | MA | One-family house detached |
| San Francisco | CA | Low rise apartment |
| Aliquippa | PA | One-family house detached |
| Albany | CA | One-family house detached |
| West Burlington | IA | One-family house detached |
| Harrisburg | IL | One-family house detached |
| Reston | VA | Low rise apartment |
| Hackettstown | NJ | One-family house attached |
| West Seneca | NY | One-family house detached |
| Morgantown | WV | One-family house attached |
| Chapel Hill | NC | One-family house detached |
| Woburn | MA | One-family house detached |
| Leavenworth | KS | One-family house detached |
| Cranberry Twp. | PA | One-family house detached |
| Lexington Park | MD | One-family house attached |
